# Connecting West and East

**DOI:** 10.3390/ijms20092333

**Published:** 2019-05-11

**Authors:** Ming Zhang, Mohamed Moalin, Guido R.M.M. Haenen

**Affiliations:** 1 Department of Pharmacology and Toxicology, Faculty of Health, Medicine and Life Sciences, Maastricht University, P.O. Box 616, 6200 MD Maastricht, The Netherlands; z.ming@maastrichtuniversity.nl (M.Z.); m.moalin@maastrichtuniversity.nl (M.M.); 2 Zuyd University of Applied Science, 6200 AN Heerlen, The Netherlands

**Keywords:** Western medicine, Eastern medicine, traditional Chinese medicine, paradigm, energy, redox modulation

## Abstract

Despite their similarities, Western medicine and Eastern medicine are very different because they are built on different fundamentals. The general idea has arisen that we will benefit by connecting Western and Eastern medicine. First, both the merits as well as the limitations of both types of medicine are discussed. It was concluded that to create a bridge, we should focus on similarities that inspire the further unravelling of the molecular mechanism of the mode of action and toxicity of Traditional Chinese Medicine. It is suggested that the energy perspective provides a basis to integrate Eastern and Western medicine.

The number one desire of most people is to be in good health. This is also reflected by the high social status of medical professionals in all societies. In most cultures, the medicine woman/man has her/his own “uniform”, uses a jargon that is not easily understood by others, and specific symbols are used (Figure 1).

Despite these similarities, medicine has been developed differently in various cultures. For example, Western medicine is quite different when compared to Eastern medicine. These have diverged because they are built on different fundamental principles. Both have their merits as well as their limitations. The general idea that has now arisen is that both types of medicine are complementary and that we could benefit by connecting Western and Eastern medicine to obtain the best of both worlds [1,2]. This is in line with the “Global Strategy and Plan of Action on Public Health, Innovation and Intellectual Property”, adopted at the 61^st^ World Health Assembly in 2008, which states that traditional medicine should be further developed by the stimulation of research and innovation [3]. This means, for example, that we need to further unravel the molecular mechanism of the mode of action and toxicity of Traditional Chinese Medicine (TCM) to try to connect TCM with Western medicine.

## 1. The Fundamentals of Western Medicine

**Figure 2:**
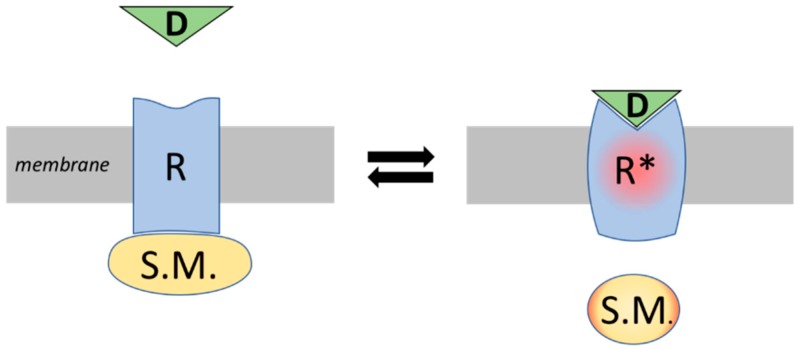
The drug-receptor concept in Western science. The paradigm is “Corpora non agunt nisi fixate”, a drug (D) will not work unless it is bound to a specific target, e.g., a receptor (R). Binding of the drug to the receptor will cause a conformational change of the structure of the receptor (R*). This triggers the production of a second messenger (S.M.), which will finally result in a strong effect in the cell.

Western medicine is “evidence based”, which means that all treatments are rational: from the molecular level to the final therapeutic effect on patients. It has evolved from the use of herbs, to the isolation and use of the most active ingredient in herbs, to the use of (semi-)synthetic compounds [4,5]. Relatively recently acquired knowledge on the chemical structure of drugs and on specific targets, e.g., receptors, has made it possible to elucidate the molecular mode of action (Figure 2) [6,7], which has revolutionized Western medicine. One of the biggest achievements of Western medicine has been the development of antibiotics, which have saved the lives of millions of people. The goal in drug development is to increase activity and specificity, and to design drugs that have a strong, specific, and immediate effect. The effects of the drugs are tested in clinical trials, which are performed according to very strict guidelines. This finally results in evidence based, standardized guidelines for the diagnosis and treatment of diseases [8,9].

## 2. The Fundamentals of Eastern Medicine

Eastern medicine has an intuitive fundamental principle. TCM has been developed in China over centuries and it is still practiced according to this old tradition [10]. Eastern medicine has been one of the driving forces of the rise of Chinese culture. In TCM, herbs are combined in a specific proportion. The concept is that herbs work together to obtain the best response. There are no specific targets. In general, the effects of TCM are relatively soft and it takes a long time to cure a disease. However, the side effects of TCM are low [11,12]. In TCM, patients are treated in a “personalized” way according to the tradition and to the doctor’s experience [13].

## 3. The Limitations of Western Medicine

From a Western point of view, there is no doubt that the “scientific” Western approach is far superior to the Eastern “intuitive” approach. However, numerous examples indicate that in the West a more humble attitude is more suitable. One of them is the unexplained effect of paracetamol. Although paracetamol is the most widely used drug and has been applied over several decades, its molecular mode of action has not been elucidated [14]. This indicates that the mysterious “placebo” effect contributes to the therapeutic effect of paracetamol. Another aspect is that many Western drugs fail to be effective in the long run. Compared to TCM, Western drugs have a very short half-life [15,16]. Even antibiotics tend to lose their effectiveness, as microorganisms adapt by becoming resistant. Moreover, the effects of Western drugs are often not as specific as proclaimed, and they do not act on the single receptor aimed for. For example, many drugs display some anticholinergic activity as a side effect. When several of these drugs are given to a patient, this might give rise to “anticholinergic accumulation” [11,17]. In addition, critical examination indicates that many controlled clinical trials, the fundamental of evidence based medicine, may actually give false results [7,18]. It is becoming apparent that most scientific findings, even if they appear to be absolutely true, could be incorrect [19]. At the best, Western science is based on axioms, but often this is only on paradigms, presumptions, prejudice, or ultimately hubris. Moreover, most pharmaceutical industries aim to generate as much money as possible, and their primary interest is not the welfare of the patient. Fraud and the manipulation of scientists and doctors are not uncommon in Western medicine. The pharmaceutical industry has even been accused of inventing and spreading diseases with the single goal to market their “miracle cure” (e.g., ritalin) [20,21].

## 4. The Limitations of Eastern medicine

**Figure 3:**
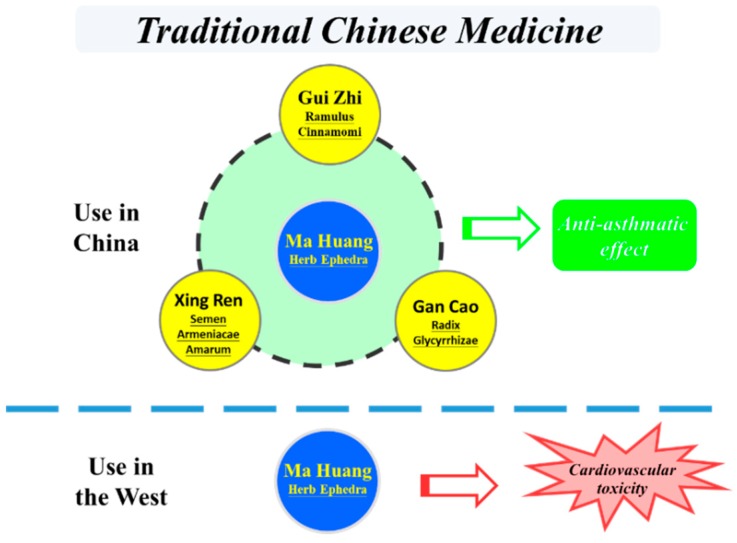
In TCM, herbs are combined together in a specific proportion to treat disease efficiently with low side effects. This is exemplified with the herb formula that contains Ma Huang as the most effect herb to treat asthma. In the West, Ma Huang is used alone and in relatively high doses, which is prone to cardiovascular side effects.

The molecular mechanisms of TCM are lacking. For example, there is no scientific proof for the existence of meridians [22]. This means that in the West, the fundamentals of TCM are denied and East and West are not connected. Nevertheless, it is increasingly apparent that TCM is valuable for also treating diseases in Western societies, especially when synthetically designed drugs fail to be effective. One of the problems is that the use of TCM has led to adverse effects in the West [5,23]. For example, Ma Huang is a herb that has been used over the centuries in China to treat asthma. By combining it with other herbs and using a relative low dose in TCM, the side effects caused by Ma Huang are reduced to a relatively safe level. However, in the USA, Ma Huang has not been used in combination with other herbs, and is used in a relatively high dose with the aim to lose weight or to act as a stimulant. This improper use of Ma Huang has given rise to serious cardiovascular problems (Figure 3) [24,25,26]. This is in line with the Western strategy to aim for a strong and quick effect, and has led to the improper use of TCM, causing side effects. This has given TCM a bad reputation and has hampered the correct appreciation of TCM. To give TCM a “scientific” basis and to elucidate its molecular mechanism, Western science needs to study TCM in the way it is supposed to be used. Moreover, Western scientists should be more open minded to the concepts of Eastern medicine and accept that the West can learn from the East.

## 5. How to Connect Western and Eastern Medicine

**Figure 4:**
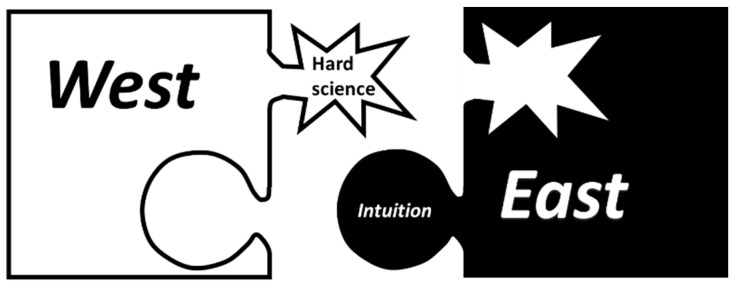
We should connect West and East to obtain a more complete picture.

Despite the diversity, the universal principle of medicine found in all cultures is that opposing forces provide the energy that flows through networks in an organism, which fuels life. In this concept, health is the ability of an organism to maintain the balance between these opposing forces, i.e., homeostasis (West) and harmony (East), which creates resilience. Additionally, therapeutic interventions are similar, namely adjusting the flow of energy by changing the connections in the network. Applying this concept opens new avenues for treatments, and gives unexpected and counterintuitive results that do not fit in the drug-receptor paradigm [11,25]. As concluded previously [27], there are numerous mysterious “forces” in Eastern medicine and other types of traditional medicine that lack a “Western scientific basis” [28], and should be studied. In doing so, Western scientists should keep an open mind. By connecting West and East, we can obtain a more complete picture (Figure 4).

## Figures and Tables

**Figure 1:**
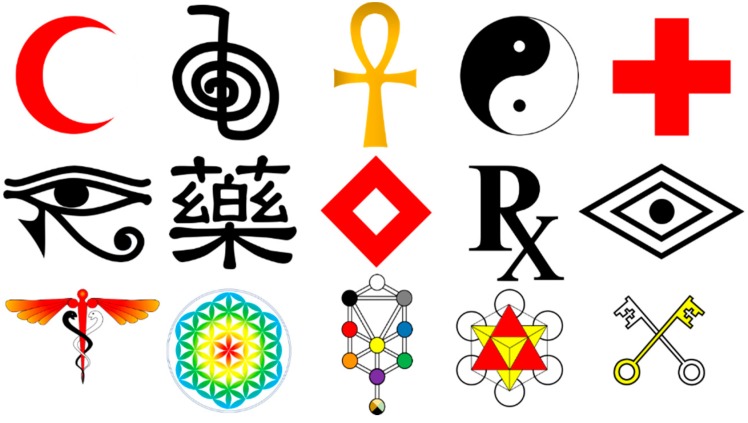
Symbols of health, medicine, and life in several cultures.

## Abbreviations

TCM	Traditional Chinese Medicine
